# Diaqua­(2,9-dimethyl-1,10-phenanthroline-κ^2^ *N*,*N*′)(4-hydroxy­benzoato-κ^2^ *O*,*O*′)cobalt(II) nitrate dihydrate

**DOI:** 10.1107/S1600536808038117

**Published:** 2008-11-22

**Authors:** Cuiping Zhai, Fengmei Yan, Peizheng Zhao

**Affiliations:** a College of Chemistry and Chemical Engineering, Henan University, Kaifeng 475001, People’s Republic of China; b Department of Chemistry and Chemical Engineering, Huanghuai University, Zhumadian 463000, People’s Republic of China; c College of Chemistry and Environmental Science, Henan Normal University, Xinxiang 453007, People’s Republic of China

## Abstract

In the title compound, [Co(C_7_H_5_O_3_)(C_14_H_12_N_2_)(H_2_O)_2_]NO_3_·2H_2_O, the Co^II^ ion is six-coordinated by two N atoms of a 2,9-dimethyl-1,10-phenanthroline (dmphen) ligand, two carboxyl­ate O atoms of one 4-hydroxy­benzoate anion and two O atoms of two water mol­ecules, in a distorted octa­hedral environment; the two water mol­ecules occupy the apical positions. In the crystal structure, the ionic units and water mol­ecules are linked through O—H⋯O hydrogen bonds, leading to the formation of a three-dimensional network. In addition, π–π inter­actions between a pyridine ring of the dmphen ligand and the benzene ring of the hydroxy­benzoate anion [centroid–centroid separation = 3.6861 (3) Å] are observed.

## Related literature

For related structures, see: Xuan *et al.* (2007 ▶); Xuan & Zhao (2007*a* ▶,*b* ▶).
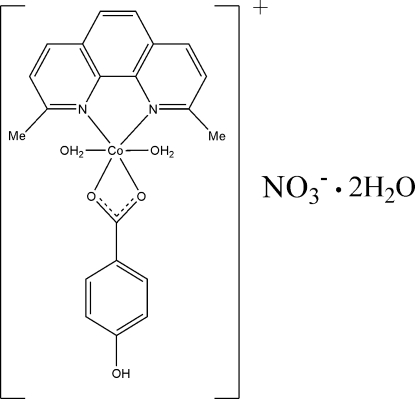


## Experimental

### 

#### Crystal data

[Co(C_7_H_5_O_3_)(C_14_H_12_N_2_)(H_2_O)_2_]NO_3_·2H_2_O
*M* *_r_* = 538.37
Monoclinic, 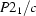

*a* = 9.8001 (8) Å
*b* = 22.2638 (19) Å
*c* = 10.8676 (9) Å
β = 94.602 (1)°
*V* = 2363.5 (3) Å^3^
*Z* = 4
Mo *K*α radiation
μ = 0.79 mm^−1^
*T* = 291 (2) K
0.35 × 0.25 × 0.14 mm

#### Data collection

Buker SMART CCD area-detector diffractometer
Absorption correction: multi-scan (*SADABS*; Bruker, 1997 ▶) *T*_min_ = 0.771, *T*_max_ = 0.899
17391 measured reflections
4385 independent reflections
3689 reflections with *I* > 2σ(*I*)
*R*_int_ = 0.024

#### Refinement

*R*[*F* ^2^ > 2σ(*F* ^2^)] = 0.029
*wR*(*F* ^2^) = 0.081
*S* = 1.01
4385 reflections
319 parameters
H-atom parameters constrained
Δρ_max_ = 0.36 e Å^−3^
Δρ_min_ = −0.24 e Å^−3^

### 

Data collection: *SMART* (Bruker, 1997 ▶); cell refinement: *SAINT* (Bruker, 1997 ▶); data reduction: *SAINT*; program(s) used to solve structure: *SHELXS97* (Sheldrick, 2008 ▶); program(s) used to refine structure: *SHELXL97* (Sheldrick, 2008 ▶); molecular graphics: *SHELXTL* (Sheldrick, 2008 ▶); software used to prepare material for publication: *publCIF* (Westrip, 2008 ▶).

## Supplementary Material

Crystal structure: contains datablocks I, global. DOI: 10.1107/S1600536808038117/ci2703sup1.cif

Structure factors: contains datablocks I. DOI: 10.1107/S1600536808038117/ci2703Isup2.hkl

Additional supplementary materials:  crystallographic information; 3D view; checkCIF report

## Figures and Tables

**Table 1:** Selected bond lengths (Å)

Co1—O5	2.0685 (14)
Co1—O4	2.1187 (14)
Co1—N1	2.1213 (15)
Co1—N2	2.1357 (15)
Co1—O1	2.1425 (13)
Co1—O2	2.2311 (13)

**Table 2:** Hydrogen-bond geometry (Å, °)

*D*—H⋯*A*	*D*—H	H⋯*A*	*D*⋯*A*	*D*—H⋯*A*
O10—H8*W*⋯O6	0.83	2.26	2.960 (3)	142
O9—H5*W*⋯O6	0.83	2.09	2.904 (3)	169
O9—H6*W*⋯O7^i^	0.83	2.09	2.888 (3)	161
O10—H7*W*⋯O8^ii^	0.83	2.01	2.829 (3)	169
O5—H4*W*⋯O10	0.81	1.93	2.720 (2)	167
O4—H2*W*⋯O2^iii^	0.83	2.01	2.846 (2)	180
O5—H3*W*⋯O1^iv^	0.82	2.05	2.826 (2)	157
O4—H1*W*⋯O9^v^	0.82	1.96	2.758 (2)	164
O3—H3⋯O8^vi^	0.82	2.57	3.133 (3)	127
O3—H3⋯O7^vi^	0.82	2.07	2.861 (3)	164

Symmetry codes: (i) 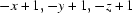; (ii) 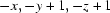; (iii) 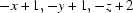; (iv) 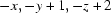; (v) 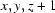; (vi) 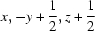.

**Fig. 1.**
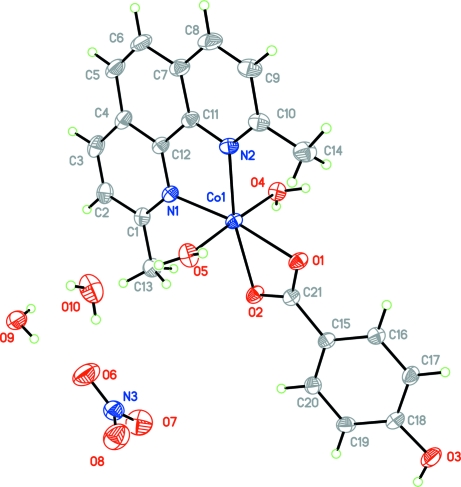
The molecular structure of the title compound, with atom labels and 30% probability displacement ellipsoids.

**Fig. 2.**
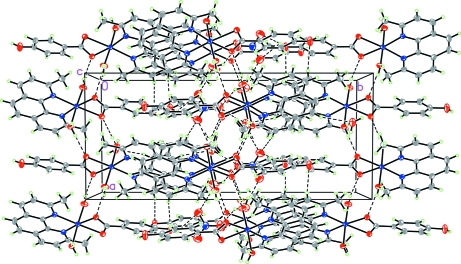
The crystal structure of the title compound, viewed along the c axis. Hydrogen bonds are shown as dashed lines.

## supplementary crystallographic information

### Comment

We have recently reported the syntheses and crystal structures of [Ni(dmphen)(3-OH-benzoate)(H_2_O)NO_3_] (Xuan & Zhao, 2007*a*), [Ni(dmphen)(benzoate)(H_2_O)NO_3_] (Xuan *et al.*,  2007) and [Co(dmphen)(3-OH-benzoate)(H_2_O)NO_3_](Xuan & Zhao, 2007*b*) complexes. Now, we report here the crystal structure of the title compound.

Each Co^II^ ion is six-coordinated by two N atoms from a dmphen ligand, and two O atoms from two water molecules and two O atoms from carboxylate group of one 4-hydroxy-benzoate anion (Fig.1). The CoO_4_N_2_ unit forms a distorted octahedral geometry, with two O atoms of two water molecules occupying the axial positions at 2.0685 (14) or 2.1187 (14) Å (Table 1). The equatorial plane is defined by the N atoms of dmphen and carboxy O atoms of the 3-hydroxybenzoate anion.

In the crystal structure, an extensive series of O—H···O hydrogen bonds, involving the coordinated and solvent water molecules, 4-hydroxybenzoate and nitrate anions, lead to a supramolecular network structure (Table 2 and Fig. 2). In addition, inversion related molecules are linked via π-π interactions involving the pyridine ring of the dmphen (N1/C1-C4/C12; centroid *Cg*1) ligand and the benzene ring of the hydroxybenzoate (C15—C20; centroid *Cg*2) anion (Fig.3); the *Cg*1—*Cg*2^iii^ distance is 3.6861 (11) Å (symmetry code is given in Table 2). This combination of hydrogen bonds and stacking interactions build a three-dimensional network.

### Experimental

To a solution of 2,9-dimethyl-1,10-phenanthroline (0.1095 g, 0.5 mmol), 4-hydroxy-benzoate (0.1382 g, 1 mmol) and sodium hydroxide (0.03740 g, 1 mmol) in ethanol-water (*v*:*v* 1:1, 15 ml) was added a solution of Ni(NO_3_)_2_.6H_2_O (0.1457 g, 0.5 mmol) in distilled water (10 ml). The resulting solution was stirred for 5 h at 323 K and then the precipitate obtained was filtered. Pink single crystals of the title compound were obtained by slow evaporation of the filtrate over 80 d.

### Refinement

C-bound H atoms were placed in calculated positions and were included in the refinement in the riding-model approximation, with C-H distances of 0.93 or 0.96 Å and *U*_iso_(H) values of 1.2 or 1.5 times *U*_eq_(C). The hydroxyl H atom was also placed in the calculated position (O-H = 0.82 Å) and refined with free torsion angles to fit the electron density. Water H atoms were located in a difference Fourier map and were allowed to ride on the parent atoms. For all O-bound H atoms the *U*_iso_(H) values were set at 1.5 *U*_eq_(O).

### Crystal data

**Table:**

[Co(C_7_H_5_O_3_)(C_14_H_12_N_2_)(H_2_O)_2_]NO_3_·2H_2_O	*F*_000_ = 1116
*M**_r_* = 538.37	*D*_x_ = 1.513 Mg m^−^^3^
Monoclinic, *P*2_1_/*c*	Mo *K*α radiation λ = 0.71073 Å
Hall symbol: -P 2ybc	Cell parameters from 6780 reflections
*a* = 9.8001 (8) Å	θ = 2.3–27.4º
*b* = 22.2638 (19) Å	µ = 0.79 mm^−^^1^
*c* = 10.8676 (9) Å	*T* = 291 (2) K
β = 94.602 (1)º	Block, pink
*V* = 2363.5 (3) Å^3^	0.35 × 0.25 × 0.14 mm
*Z* = 4	

### Data collection

**Table:**

Buker SMART CCD area-detector diffractometer	4385 independent reflections
Radiation source: fine-focus sealed tube	3689 reflections with *I* > 2σ(*I*)
Monochromator: graphite	*R*_int_ = 0.024
*T* = 291(2) K	θ_max_ = 25.5º
φ and ω scans	θ_min_ = 2.3º
Absorption correction: multi-scan(SADABS; Bruker, 1997)	*h* = −11→11
*T*_min_ = 0.771, *T*_max_ = 0.899	*k* = −26→26
17391 measured reflections	*l* = −12→13

### Refinement

**Table:**

Refinement on *F*^2^	Hydrogen site location: inferred from neighbouring sites
Least-squares matrix: full	H-atom parameters constrained
*R*[*F*^2^ > 2σ(*F*^2^)] = 0.029	*w* = 1/[σ^2^(*F*_o_^2^) + (0.0425*P*)^2^ + 0.9518*P*] where *P* = (*F*_o_^2^ + 2*F*_c_^2^)/3
*wR*(*F*^2^) = 0.081	(Δ/σ)_max_ = 0.002
*S* = 1.01	Δρ_max_ = 0.36 e Å^−^^3^
4385 reflections	Δρ_min_ = −0.23 e Å^−^^3^
319 parameters	Extinction correction: SHELXL97 (Sheldrick, 2008), Fc^*^=kFc[1+0.001xFc^2^λ^3^/sin(2θ)]^-1/4^
Primary atom site location: structure-invariant direct methods	Extinction coefficient: 0.0047 (4)
Secondary atom site location: difference Fourier map	

### Special details

**Table:**

Geometry. All e.s.d.'s (except the e.s.d. in the dihedral angle between two l.s. planes)are estimated using the full covariance matrix. The cell e.s.d.'s are takeninto account individually in the estimation of e.s.d.'s in distances, anglesand torsion angles; correlations between e.s.d.'s in cell parameters are onlyused when they are defined by crystal symmetry. An approximate (isotropic)treatment of cell e.s.d.'s is used for estimating e.s.d.'s involving l.s. planes.
Refinement. Refinement of *F*^2^ against ALL reflections. The weighted *R*-factor *wR* andgoodness of fit *S* are based on *F*^2^, conventional *R*-factors *R* are basedon *F*, with *F* set to zero for negative *F*^2^. The threshold expression of*F*^2^ > σ(*F*^2^) is used only for calculating *R*-factors(gt) *etc*. and isnot relevant to the choice of reflections for refinement. *R*-factors basedon *F*^2^ are statistically about twice as large as those based on *F*, and *R*-factors based on ALL data will be even larger.

### Fractional atomic coordinates and isotropic or equivalent isotropic displacement parameters (Å^2^)

**Table:**

	*x*	*y*	*z*	*U*_iso_*/*U*_eq_	
Co1	0.24510 (2)	0.564923 (11)	0.97828 (2)	0.02979 (10)	
O1	0.17733 (13)	0.49074 (6)	1.08320 (12)	0.0366 (3)	
O2	0.33021 (13)	0.47337 (6)	0.95100 (12)	0.0374 (3)	
O3	0.2669 (2)	0.21307 (7)	1.18959 (16)	0.0605 (5)	
H3	0.2922	0.1902	1.1368	0.091*	
O4	0.41725 (13)	0.57880 (6)	1.10585 (13)	0.0393 (3)	
H1W	0.3985	0.5685	1.1749	0.059*	
H2W	0.4911	0.5635	1.0890	0.059*	
O5	0.08164 (14)	0.54783 (7)	0.85063 (13)	0.0463 (4)	
H3W	0.0120	0.5431	0.8866	0.070*	
H4W	0.0724	0.5513	0.7766	0.070*	
O6	0.2587 (2)	0.46630 (9)	0.4839 (2)	0.0894 (7)	
O7	0.3605 (2)	0.38408 (10)	0.5460 (2)	0.0809 (6)	
O8	0.1450 (2)	0.38603 (9)	0.5079 (2)	0.0793 (6)	
N1	0.32511 (15)	0.62761 (7)	0.85585 (14)	0.0318 (3)	
N2	0.15796 (15)	0.64623 (7)	1.04080 (15)	0.0335 (4)	
N3	0.2548 (2)	0.41299 (10)	0.51267 (18)	0.0539 (5)	
C1	0.40177 (19)	0.61783 (9)	0.76179 (18)	0.0365 (4)	
C2	0.4510 (2)	0.66576 (10)	0.6932 (2)	0.0453 (5)	
H2	0.5028	0.6578	0.6270	0.054*	
C3	0.4234 (2)	0.72318 (10)	0.7231 (2)	0.0488 (5)	
H3A	0.4580	0.7547	0.6788	0.059*	
C4	0.3424 (2)	0.73521 (9)	0.8210 (2)	0.0411 (5)	
C5	0.3063 (2)	0.79459 (9)	0.8560 (2)	0.0508 (6)	
H5A	0.3428	0.8274	0.8170	0.061*	
C6	0.2207 (3)	0.80379 (9)	0.9444 (2)	0.0522 (6)	
H6A	0.1975	0.8428	0.9649	0.063*	
C7	0.1645 (2)	0.75432 (9)	1.0074 (2)	0.0425 (5)	
C8	0.0722 (2)	0.76153 (10)	1.0991 (2)	0.0523 (6)	
H8A	0.0427	0.7997	1.1195	0.063*	
C9	0.0264 (2)	0.71247 (10)	1.1575 (2)	0.0495 (6)	
H9	−0.0353	0.7172	1.2176	0.059*	
C10	0.07125 (19)	0.65450 (9)	1.12827 (19)	0.0393 (5)	
C11	0.20282 (19)	0.69525 (8)	0.98025 (18)	0.0343 (4)	
C12	0.29325 (18)	0.68556 (8)	0.88414 (18)	0.0334 (4)	
C13	0.4357 (2)	0.55479 (10)	0.7305 (2)	0.0475 (5)	
H13A	0.5202	0.5434	0.7752	0.071*	
H13B	0.4450	0.5517	0.6435	0.071*	
H13C	0.3638	0.5286	0.7527	0.071*	
C14	0.0247 (2)	0.60129 (10)	1.1968 (2)	0.0500 (6)	
H14A	−0.0272	0.5751	1.1406	0.075*	
H14B	−0.0317	0.6144	1.2599	0.075*	
H14C	0.1028	0.5802	1.2340	0.075*	
C15	0.25751 (18)	0.38985 (8)	1.06962 (18)	0.0315 (4)	
C16	0.21346 (19)	0.37361 (8)	1.18382 (18)	0.0350 (4)	
H16	0.1813	0.4030	1.2350	0.042*	
C17	0.2169 (2)	0.31459 (9)	1.22188 (19)	0.0398 (5)	
H17	0.1887	0.3044	1.2989	0.048*	
C18	0.2626 (2)	0.27028 (9)	1.1450 (2)	0.0401 (5)	
C19	0.3045 (2)	0.28577 (9)	1.03029 (19)	0.0408 (5)	
H19	0.3334	0.2561	0.9781	0.049*	
C20	0.3033 (2)	0.34506 (9)	0.99351 (19)	0.0374 (4)	
H20	0.3333	0.3552	0.9172	0.045*	
C21	0.25521 (18)	0.45407 (9)	1.03250 (17)	0.0326 (4)	
O9	0.38611 (19)	0.56246 (7)	0.35357 (16)	0.0590 (4)	
H5W	0.3520	0.5320	0.3824	0.089*	
H6W	0.4632	0.5696	0.3884	0.089*	
O10	0.0934 (2)	0.55782 (10)	0.60222 (17)	0.0801 (6)	
H7W	0.0195	0.5697	0.5695	0.120*	
H8W	0.1089	0.5228	0.5809	0.120*	

### Atomic displacement parameters (Å^2^)

**Table:**

	*U*^11^	*U*^22^	*U*^33^	*U*^12^	*U*^13^	*U*^23^
Co1	0.02697 (15)	0.02584 (15)	0.03723 (16)	0.00085 (9)	0.00663 (10)	0.00142 (10)
O1	0.0339 (7)	0.0294 (7)	0.0479 (8)	0.0028 (5)	0.0117 (6)	0.0027 (6)
O2	0.0354 (7)	0.0328 (7)	0.0457 (8)	−0.0002 (6)	0.0131 (6)	0.0061 (6)
O3	0.0890 (13)	0.0308 (8)	0.0644 (11)	0.0015 (8)	0.0237 (10)	0.0087 (7)
O4	0.0313 (7)	0.0439 (8)	0.0428 (8)	0.0022 (6)	0.0042 (6)	0.0025 (6)
O5	0.0323 (7)	0.0644 (10)	0.0423 (8)	−0.0086 (7)	0.0028 (6)	0.0030 (7)
O6	0.123 (2)	0.0482 (12)	0.1012 (16)	−0.0095 (11)	0.0364 (15)	0.0098 (10)
O7	0.0641 (12)	0.0851 (14)	0.0940 (16)	0.0112 (11)	0.0088 (11)	−0.0044 (12)
O8	0.0701 (13)	0.0730 (13)	0.0944 (15)	−0.0149 (11)	0.0030 (11)	0.0118 (11)
N1	0.0275 (8)	0.0300 (8)	0.0381 (9)	0.0009 (6)	0.0030 (6)	0.0036 (7)
N2	0.0275 (8)	0.0311 (8)	0.0421 (9)	0.0027 (6)	0.0030 (7)	−0.0035 (7)
N3	0.0643 (14)	0.0494 (12)	0.0491 (12)	−0.0014 (10)	0.0115 (10)	−0.0038 (9)
C1	0.0293 (10)	0.0426 (11)	0.0375 (11)	0.0018 (8)	0.0022 (8)	0.0050 (9)
C2	0.0391 (11)	0.0553 (14)	0.0422 (12)	−0.0014 (10)	0.0080 (9)	0.0113 (10)
C3	0.0433 (12)	0.0488 (13)	0.0542 (14)	−0.0077 (10)	0.0030 (10)	0.0193 (11)
C4	0.0393 (11)	0.0342 (11)	0.0484 (12)	−0.0040 (8)	−0.0049 (9)	0.0097 (9)
C5	0.0586 (14)	0.0299 (11)	0.0622 (15)	−0.0052 (10)	−0.0047 (12)	0.0104 (10)
C6	0.0642 (15)	0.0247 (10)	0.0656 (16)	0.0058 (10)	−0.0087 (12)	0.0015 (10)
C7	0.0411 (12)	0.0333 (11)	0.0516 (13)	0.0077 (9)	−0.0055 (9)	−0.0022 (9)
C8	0.0513 (13)	0.0366 (12)	0.0684 (16)	0.0153 (10)	0.0008 (11)	−0.0092 (11)
C9	0.0425 (12)	0.0484 (13)	0.0589 (14)	0.0131 (10)	0.0111 (10)	−0.0110 (11)
C10	0.0298 (10)	0.0410 (11)	0.0472 (12)	0.0036 (8)	0.0041 (8)	−0.0066 (9)
C11	0.0306 (9)	0.0285 (10)	0.0426 (11)	0.0013 (8)	−0.0044 (8)	0.0010 (8)
C12	0.0277 (9)	0.0306 (10)	0.0411 (11)	0.0005 (7)	−0.0028 (8)	0.0034 (8)
C13	0.0509 (13)	0.0468 (12)	0.0471 (13)	0.0047 (10)	0.0180 (10)	0.0009 (10)
C14	0.0475 (13)	0.0481 (13)	0.0576 (14)	0.0002 (10)	0.0235 (11)	−0.0060 (11)
C15	0.0272 (9)	0.0291 (9)	0.0385 (10)	−0.0024 (7)	0.0041 (8)	0.0015 (8)
C16	0.0323 (10)	0.0337 (10)	0.0396 (11)	0.0005 (8)	0.0071 (8)	−0.0011 (8)
C17	0.0419 (11)	0.0371 (11)	0.0416 (11)	−0.0036 (9)	0.0109 (9)	0.0064 (9)
C18	0.0424 (11)	0.0292 (10)	0.0489 (12)	−0.0045 (8)	0.0041 (9)	0.0052 (9)
C19	0.0455 (11)	0.0324 (10)	0.0453 (12)	−0.0011 (9)	0.0082 (9)	−0.0059 (9)
C20	0.0389 (11)	0.0373 (11)	0.0369 (11)	−0.0017 (9)	0.0090 (8)	0.0007 (9)
C21	0.0283 (9)	0.0324 (10)	0.0373 (11)	−0.0018 (8)	0.0028 (8)	0.0009 (8)
O9	0.0772 (12)	0.0438 (9)	0.0572 (10)	0.0025 (8)	0.0119 (9)	0.0023 (7)
O10	0.0746 (13)	0.1064 (17)	0.0584 (12)	0.0092 (11)	0.0001 (10)	−0.0031 (11)

### Geometric parameters (Å, °)

**Table:**

Co1—O5	2.0685 (14)	C6—C7	1.430 (3)
Co1—O4	2.1187 (14)	C6—H6A	0.93
Co1—N1	2.1213 (15)	C7—C11	1.406 (3)
Co1—N2	2.1357 (15)	C7—C8	1.407 (3)
Co1—O1	2.1425 (13)	C8—C9	1.358 (3)
Co1—O2	2.2311 (13)	C8—H8A	0.93
O1—C21	1.273 (2)	C9—C10	1.408 (3)
O2—C21	1.270 (2)	C9—H9	0.93
O3—C18	1.362 (2)	C10—C14	1.490 (3)
O3—H3	0.82	C11—C12	1.439 (3)
O4—H1W	0.82	C13—H13A	0.96
O4—H2W	0.83	C13—H13B	0.96
O5—H3W	0.82	C13—H13C	0.96
O5—H4W	0.81	C14—H14A	0.96
O6—N3	1.229 (3)	C14—H14B	0.96
O7—N3	1.249 (3)	C14—H14C	0.96
O8—N3	1.229 (3)	C15—C20	1.392 (3)
N1—C1	1.334 (2)	C15—C16	1.394 (3)
N1—C12	1.368 (2)	C15—C21	1.485 (3)
N2—C10	1.338 (2)	C16—C17	1.377 (3)
N2—C11	1.365 (2)	C16—H16	0.93
C1—C2	1.409 (3)	C17—C18	1.390 (3)
C1—C13	1.488 (3)	C17—H17	0.93
C2—C3	1.352 (3)	C18—C19	1.386 (3)
C2—H2	0.93	C19—C20	1.379 (3)
C3—C4	1.403 (3)	C19—H19	0.93
C3—H3A	0.93	C20—H20	0.93
C4—C12	1.407 (3)	O9—H5W	0.83
C4—C5	1.428 (3)	O9—H6W	0.83
C5—C6	1.341 (3)	O10—H7W	0.82
C5—H5A	0.93	O10—H8W	0.83
			
O5—Co1—O4	177.28 (6)	C11—C7—C6	120.0 (2)
O5—Co1—N1	90.33 (6)	C8—C7—C6	123.0 (2)
O4—Co1—N1	89.97 (6)	C9—C8—C7	119.7 (2)
O5—Co1—N2	93.52 (6)	C9—C8—H8A	120.2
O4—Co1—N2	89.19 (6)	C7—C8—H8A	120.2
N1—Co1—N2	79.59 (6)	C8—C9—C10	120.7 (2)
O5—Co1—O1	87.63 (6)	C8—C9—H9	119.7
O4—Co1—O1	91.65 (5)	C10—C9—H9	119.7
N1—Co1—O1	170.71 (6)	N2—C10—C9	120.96 (19)
N2—Co1—O1	109.58 (6)	N2—C10—C14	118.91 (17)
O5—Co1—O2	91.17 (6)	C9—C10—C14	120.12 (19)
O4—Co1—O2	86.19 (5)	N2—C11—C7	123.02 (19)
N1—Co1—O2	110.90 (5)	N2—C11—C12	118.08 (16)
N2—Co1—O2	168.50 (6)	C7—C11—C12	118.90 (18)
O1—Co1—O2	60.11 (5)	N1—C12—C4	122.56 (18)
C21—O1—Co1	92.40 (11)	N1—C12—C11	118.00 (16)
C21—O2—Co1	88.46 (11)	C4—C12—C11	119.42 (18)
C18—O3—H3	109.5	C1—C13—H13A	109.5
Co1—O4—H1W	109.5	C1—C13—H13B	109.5
Co1—O4—H2W	117.1	H13A—C13—H13B	109.5
H1W—O4—H2W	110.5	C1—C13—H13C	109.5
Co1—O5—H3W	109.5	H13A—C13—H13C	109.5
Co1—O5—H4W	132.1	H13B—C13—H13C	109.5
H3W—O5—H4W	117.1	C10—C14—H14A	109.5
C1—N1—C12	118.60 (16)	C10—C14—H14B	109.5
C1—N1—Co1	129.23 (13)	H14A—C14—H14B	109.5
C12—N1—Co1	112.14 (12)	C10—C14—H14C	109.5
C10—N2—C11	118.69 (16)	H14A—C14—H14C	109.5
C10—N2—Co1	129.48 (13)	H14B—C14—H14C	109.5
C11—N2—Co1	111.83 (12)	C20—C15—C16	118.73 (17)
O6—N3—O8	120.4 (2)	C20—C15—C21	121.77 (17)
O6—N3—O7	122.0 (2)	C16—C15—C21	119.49 (17)
O8—N3—O7	117.6 (2)	C17—C16—C15	120.86 (18)
N1—C1—C2	121.30 (19)	C17—C16—H16	119.6
N1—C1—C13	118.61 (17)	C15—C16—H16	119.6
C2—C1—C13	120.09 (19)	C16—C17—C18	119.85 (18)
C3—C2—C1	120.4 (2)	C16—C17—H17	120.1
C3—C2—H2	119.8	C18—C17—H17	120.1
C1—C2—H2	119.8	O3—C18—C19	123.33 (19)
C2—C3—C4	119.94 (19)	O3—C18—C17	116.85 (18)
C2—C3—H3A	120.0	C19—C18—C17	119.79 (18)
C4—C3—H3A	120.0	C20—C19—C18	120.15 (19)
C3—C4—C12	117.17 (19)	C20—C19—H19	119.9
C3—C4—C5	123.1 (2)	C18—C19—H19	119.9
C12—C4—C5	119.7 (2)	C19—C20—C15	120.59 (19)
C6—C5—C4	121.0 (2)	C19—C20—H20	119.7
C6—C5—H5A	119.5	C15—C20—H20	119.7
C4—C5—H5A	119.5	O2—C21—O1	119.03 (18)
C5—C6—C7	120.8 (2)	O2—C21—C15	121.20 (17)
C5—C6—H6A	119.6	O1—C21—C15	119.77 (16)
C7—C6—H6A	119.6	H5W—O9—H6W	111.3
C11—C7—C8	116.9 (2)	H7W—O10—H8W	110.8
			
O5—Co1—O1—C21	93.01 (11)	C11—N2—C10—C9	0.2 (3)
O4—Co1—O1—C21	−84.36 (11)	Co1—N2—C10—C9	179.14 (15)
N2—Co1—O1—C21	−174.09 (11)	C11—N2—C10—C14	−178.51 (18)
O2—Co1—O1—C21	0.29 (10)	Co1—N2—C10—C14	0.4 (3)
O5—Co1—O2—C21	−86.88 (11)	C8—C9—C10—N2	−1.2 (3)
O4—Co1—O2—C21	93.81 (11)	C8—C9—C10—C14	177.5 (2)
N1—Co1—O2—C21	−177.68 (10)	C10—N2—C11—C7	1.4 (3)
N2—Co1—O2—C21	27.2 (3)	Co1—N2—C11—C7	−177.69 (15)
O1—Co1—O2—C21	−0.29 (10)	C10—N2—C11—C12	−178.40 (17)
O5—Co1—N1—C1	−83.02 (16)	Co1—N2—C11—C12	2.5 (2)
O4—Co1—N1—C1	94.28 (16)	C8—C7—C11—N2	−2.0 (3)
N2—Co1—N1—C1	−176.54 (17)	C6—C7—C11—N2	176.94 (19)
O2—Co1—N1—C1	8.36 (18)	C8—C7—C11—C12	177.86 (18)
O5—Co1—N1—C12	98.92 (13)	C6—C7—C11—C12	−3.2 (3)
O4—Co1—N1—C12	−83.79 (12)	C1—N1—C12—C4	−2.5 (3)
N2—Co1—N1—C12	5.40 (12)	Co1—N1—C12—C4	175.82 (14)
O2—Co1—N1—C12	−169.70 (11)	C1—N1—C12—C11	175.81 (17)
O5—Co1—N2—C10	87.09 (17)	Co1—N1—C12—C11	−5.9 (2)
O4—Co1—N2—C10	−93.10 (17)	C3—C4—C12—N1	2.1 (3)
N1—Co1—N2—C10	176.78 (18)	C5—C4—C12—N1	−179.36 (18)
O1—Co1—N2—C10	−1.64 (18)	C3—C4—C12—C11	−176.18 (18)
O2—Co1—N2—C10	−26.8 (4)	C5—C4—C12—C11	2.4 (3)
O5—Co1—N2—C11	−93.90 (13)	N2—C11—C12—N1	2.4 (3)
O4—Co1—N2—C11	85.91 (13)	C7—C11—C12—N1	−177.50 (17)
N1—Co1—N2—C11	−4.21 (12)	N2—C11—C12—C4	−179.31 (17)
O1—Co1—N2—C11	177.37 (12)	C7—C11—C12—C4	0.8 (3)
O2—Co1—N2—C11	152.2 (2)	C20—C15—C16—C17	1.0 (3)
C12—N1—C1—C2	0.8 (3)	C21—C15—C16—C17	−178.71 (17)
Co1—N1—C1—C2	−177.17 (14)	C15—C16—C17—C18	−1.1 (3)
C12—N1—C1—C13	−179.64 (17)	C16—C17—C18—O3	178.27 (19)
Co1—N1—C1—C13	2.4 (3)	C16—C17—C18—C19	0.0 (3)
N1—C1—C2—C3	1.2 (3)	O3—C18—C19—C20	−176.9 (2)
C13—C1—C2—C3	−178.3 (2)	C17—C18—C19—C20	1.2 (3)
C1—C2—C3—C4	−1.6 (3)	C18—C19—C20—C15	−1.4 (3)
C2—C3—C4—C12	0.0 (3)	C16—C15—C20—C19	0.2 (3)
C2—C3—C4—C5	−178.5 (2)	C21—C15—C20—C19	179.93 (18)
C3—C4—C5—C6	175.1 (2)	Co1—O2—C21—O1	0.49 (17)
C12—C4—C5—C6	−3.3 (3)	Co1—O2—C21—C15	−179.24 (16)
C4—C5—C6—C7	0.9 (4)	Co1—O1—C21—O2	−0.51 (17)
C5—C6—C7—C11	2.4 (3)	Co1—O1—C21—C15	179.23 (15)
C5—C6—C7—C8	−178.8 (2)	C20—C15—C21—O2	−21.5 (3)
C11—C7—C8—C9	0.9 (3)	C16—C15—C21—O2	158.19 (18)
C6—C7—C8—C9	−178.0 (2)	C20—C15—C21—O1	158.77 (18)
C7—C8—C9—C10	0.6 (3)	C16—C15—C21—O1	−21.5 (3)

### Hydrogen-bond geometry (Å, °)

**Table:**

*D*—H···*A*	*D*—H	H···*A*	*D*···*A*	*D*—H···*A*
O10—H8W···O6	0.83	2.26	2.960 (3)	142
O9—H5W···O6	0.83	2.09	2.904 (3)	169
O9—H6W···O7^i^	0.83	2.09	2.888 (3)	161
O10—H7W···O8^ii^	0.83	2.01	2.829 (3)	169
O5—H4W···O10	0.81	1.93	2.720 (2)	167
O4—H2W···O2^iii^	0.83	2.01	2.846 (2)	180
O5—H3W···O1^iv^	0.82	2.05	2.826 (2)	157
O4—H1W···O9^v^	0.82	1.96	2.758 (2)	164
O3—H3···O8^vi^	0.82	2.57	3.133 (3)	127
O3—H3···O7^vi^	0.82	2.07	2.861 (3)	164

Symmetry codes: (i) −*x*+1, −*y*+1, −*z*+1; (ii) −*x*, −*y*+1, −*z*+1; (iii) −*x*+1, −*y*+1, −*z*+2; (iv) −*x*, −*y*+1, −*z*+2; (v) *x*, *y*, *z*+1; (vi) *x*, −*y*+1/2, *z*+1/2.

## References

[bb1] Bruker (1997). *SMART*, *SAINT* and *SADABS* Bruker AXS Inc., Madison, Wisconsin, USA.

[bb2] Sheldrick, G. M. (2008). *Acta Cryst.* A**64**, 112–122. 10.1107/S0108767307043930 18156677

[bb3] Westrip, S. P. (2008). *publCIF.* In preparation.

[bb4] Xuan, X. & Zhao, P. (2007*a*). *Acta Cryst.* E**63**, m2856.

[bb5] Xuan, X. & Zhao, P. (2007*b*). *Acta Cryst.* E**63**, m3009.

[bb6] Xuan, X.-P., Zhao, P.-Z. & Tang, Q.-H. (2007). *Acta Cryst.* E**63**, m2405.

